# Characterization of efficient xylanases from industrial-scale pulp and paper wastewater treatment microbiota

**DOI:** 10.1186/s13568-020-01178-1

**Published:** 2021-01-19

**Authors:** Jia Wang, Jiawei Liang, Yonghong Li, Lingmin Tian, Yongjun Wei

**Affiliations:** 1 Key Laboratory of Advanced Drug Preparation Technologies, Ministry of Education, School of Pharmaceutical Sciences, Zhengzhou University, Zhengzhou, People’s Republic of China; 2 College of Public Health, Zhengzhou University, Zhengzhou, Henan 450001 People’s Republic of China; 3 Department of Food Science and Engineering, Jinan University, Guangzhou, 510632 People’s Republic of China

**Keywords:** Metagenome, Xylanase, GH10 family, Xylan, Pulp and paper wastewater

## Abstract

Xylanases are widely used enzymes in the food, textile, and paper industries. Most efficient xylanases have been identified from lignocellulose-degrading microbiota, such as the microbiota of the cow rumen and the termite hindgut. Xylanase genes from efficient pulp and paper wastewater treatment (PPWT) microbiota have been previously recovered by metagenomics, assigning most of the xylanase genes to the GH10 family. In this study, a total of 40 GH10 family xylanase genes derived from a certain PPWT microbiota were cloned and expressed in *Escherichia coli* BL21 (DE3). Among these xylanase genes, 14 showed xylanase activity on beechwood substrate. Two of these, PW-xyl9 and PW-xyl37, showed high activities, and were purified to evaluate their xylanase properties. Values of optimal pH and temperature for PW-xyl9 were pH 7 and 60 ℃, respectively, while those for PW-xyl37 were pH 7 and 55 ℃, respectively; their specific xylanase activities under optimal conditions were 470.1 U/mg protein and 113.7 U/mg protein, respectively. Furthermore, the *Km* values of PW-xyl9 and PW-xyl37 were determined as 8.02 and 18.8 g/L, respectively. The characterization of these two xylanases paves the way for potential application in future pulp and paper production and other industries, indicating that PPWT microbiota has been an undiscovered reservoir of efficient lignocellulase genes. This study demonstrates that a metagenomic approach has the potential to screen efficient xylanases of uncultured microorganisms from lignocellulose-degrading microbiota. In a similar way, other efficient lignocellulase genes might be identified from PPWT treatment microbiota in the future.

## Keypoints

- Xylanases sourced from a certain pulp and paper wastewater treatment microbiota were cloned and characterized
- Two xylanases had high performance under wide pH and temperature ranges
- The potential structures of active sites of the two xylanases were identified through bioinformatics analyses

## Introduction

Xylan, a major component of hemicellulose in plant cells, is among the most abundant natural sources of renewable biomass (Scheller and Ulvskov 2010; Walia et al. 2017). It consists of xylose units connected through β-1,4-glycosidic bonds and side chains composed of α-D-glucuronide, arabinose, galactose, acetate, methyl glucuronic acid and other simple sugars (Houfani et al. 2020). The microbial degradation of xylan requires the synergy of a variety of enzymes—with xylanase as the most essential among them—which can hydrolyze β-1,4-glycosidic bonds to produce xylose or xylose oligosaccharides (Shallom and Shoham 2003). Xylanases are widely used in the paper manufacturing, textile, biofuel, food and other industries, as they can increase the efficiency of lignocellulose usage and reduce environmental pollution (Juturu and Wu 2012; Kumar et al. 2018). Previously, fungal xylanases were mainly used for the degradation of xylan from plant biomass during biofuel manufacture and other industrial applications (Torres and Dela Cruz 2013; Walia et al. 2017). Due to the diversity of naturally occurring efficient lignocellulose degradation systems, there is a possibility to recover novel types of xylanase from these for the efficient enzymatic degradation of xylan (Mhiri et al. 2020).

With the development of microbiome profiling and other high-throughput sequencing technologies, xylanases were identified in typical efficient lignocellulose degradation microbiota, such as biogas digesters, the termite hindgut, the cow rumen, and the camel gut (Gharechahi and Salekdeh 2018; Liu et al. 2019; Stewart et al. 2018; Wei et al. 2015). At least 386 different xylanase genes were screened from a single biogas digester, with xylanase activities detected by the expression of the recovered xylanase genes in *Escherichia coli* (Wei et al. 2015; Yan et al. 2013). The termite hindgut is an efficient microbial niche for lignocellulose degradation, from where certain xylanases have been identified (Liu et al. 2011, 2019). Some of these xylanases show high xylanase activity, which partly explains the reason for the high lignocellulose-degrading efficiency of the termite (Han et al. 2013; Qian et al. 2015). A total of 913 microbial genomes and 69,000 genes related to carbohydrate metabolism were identified through the assembly of cow rumen metagenomic sequences, characterizing the cow rumen as a reservoir of xylanase genes (Stewart et al. 2018). The artificial rumen system is highly efficient in degrading lignocellulose for methane production (Xing et al. 2020), harboring diverse functional xylanases (Loaces et al. 2016). Moreover, many efficient xylanase genes have been isolated from camel and kangaroo gut microbiota (Gharechahi and Salekdeh 2018; Wirth et al. 2018); some of them showed alkali-thermostable characteristics, which makes them useful in the enhancement of recalcitrant lignocellulose biomass degradation (Ariaeenejad et al. 2019; Ghadikolaei et al. 2019).

Diverse xylanases are present in most biogas digesters inoculated with lignocellulose, thus they have the ability to degrade lignocellulose efficiently (Liang et al. 2021; Sawatdeenarunat et al. 2015; Wei et al. 2015). Paper and pulp manufacture wastewater contains high levels of lignocellulose waste, and the microbiota used to treat such wastewater harbors a diversity of lignocellulose genes. In our previous study, over 93,000 genes predicted to be associated with carbohydrate metabolism were identified in the anaerobic and aerobic microbiota used for pulp and paper wastewater treatment (PPWT) (Liang et al. 2021). These xylanase genes belonged to more than 16 different glycoside hydrolase (GH) families in the CAZy database, with most enzymes of GH10 and GH11 families showing xylanase activity (Lombard et al. 2014). Most xylanase genes were assigned to the GH10 family among these genes, and more than 100 GH10 family genes or gene fragments were identified in the PPWT microbiota (Liang et al. 2021). Meanwhile, no GH11 family genes were recovered from the anaerobic microbiota, and only eleven GH11 family gene fragments were identified in the aerobic microbiota of the PPWT plant (Liang et al. 2021).

Xylanases of the GH10 family, which normally have high molecular weight and a (β/α)_8_ 8-barrel folding structure, hydrolyze xylan through a reservation-type catalytic mechanism (Kim et al. 2018; Zhang et al. 2016). Crystal structure and dynamics analysis have revealed that these xylanases have four to five active binding sites, and often contain carbohydrate-binding modules (CBM) connected to their catalytic structural domain (Jia and Han 2019; Wu et al. 2020). These CBM molecules help to improve the specific binding of xylanases to lignocellulose substrate, leading to enhanced xylan hydrolysis ability (Bhardwaj et al. 2012; Collins et al. 2005). The Xyn10J xylanase obtained from the metagenomic library of one compost microbiota can improve the saccharification of lignocellulose in biomass, and is useful for the production of fermented sugars (Jeong et al. 2012). The GH10 xylanases of efficient lignocellulose-degrading PPWT microbiota might harbor high xylanase ability, and are robust to extreme xylanase reaction environments (Liang et al. 2021).

In the current study, we attempt to characterize GH10 family xylanase genes from the efficient microbiota of a specific PPWT facility (Liang et al. 2021). Cloned full-length GH10 family genes were expressed in *E. coli*, the activities of two expressed GH10 family xylanases were characterized, and their xylanase properties were described in details.

## Materials and methods

### Strains, plasmid, and reagents

*E. coli* TOP10 and BL21 cell (DE3) strains were purchased from Tolo Biotechnology (Anhui, China). The beechwood as xylanase substrate was sourced from Megazyme Co. Ltd (www.megazyme.com). The 3,5-dinitrosalicylic acid (DNS) and other chemical reagents were obtained from China National Pharmaceutical Group Co., Ltd. (Haidian District, Beijing, China) (Hu et al. 2008).

### DNA analysis, screening and plasmid construction

In a previous study, a diverse array of CAZy genes and a total of 162 GH10 family genes were recovered from the microbiota of a particular PPWT plant by metagenomic assembly (Liang et al. 2021). In our study, the GH10 family genes that were predicted to be full-length and showed more than 25% identity with known xylanase genes in the GenBank database were selected for further cloning. Finally, a total of 40 xylanase genes were ultimately verified by Sanger sequencing, and were named as *PW-Xyl1* to *PW-Xyl40* (Table 1). These genes were aligned with certain high activity xylanase genes downloaded from the enzyme repository of the ExPaSy database at https://enzyme.expasy.org/. The phylogenetic tree of xylanase genes was constructed with MEGA 7 using the Test UPGMA tree method (Kumar et al. 2016), and was further polished with iTOL (Letunic and Bork 2019).

**Table 1:** The 40 cloned xylanase genes and their closest gene counterparts in the GenBank database

Name	AA^a^	Closest genes and their accession numbers	Identity (%)	S^b^
*PW-xyl1*	333	TPA: 1,4-β-xylanase [*Cyanobacteria* bacterium] (UBA11371) (HAZ45611.1)	55.66	
*PW-xyl2*	360	Endo-1,4-β-xylanase [uncultured bacterium Contig15] (AHF23866.1)	77.87	
*PW-xyl3*	368	Endo-1,4-β-xylanase [*Hallella seregens*] (WP_052323291.1)	75.68	
*PW-xyl4*	369	Endo-1,4-β-xylanase [Candidatus *Atribacteria* bacterium] (TFG90307.1)	73.49	✓
*PW-xyl5*	370	Endo-1,4-β-xylanase [*Ignavibacteriae* bacterium] (RPI05742.1)	51.52	✓
*PW-xyl6*	372	Endo-1,4-β-xylanase [*Ignavibacteriae* bacterium] (RPI05742.1)	53.68	
*PW-xyl7*	374	Endo-1,4-β-xylanase [*Rufibacter* sp. R-22-1c-1] (WP_123126834.1)	63.71	✓
*PW-xyl8*	374	Endo-1,4-β-xylanase [*Paludibacter jiangxiensis*] (WP_068704526.1)	81.01	✓
*PW-xyl9*	377	Endo-1,4-β-xylanase [*Bacteroidales* bacterium] (NLJ21332.1)	89.30	✓
*PW-xyl10*	383	Endo-1,4-β-xylanase [*Alkalilimnicola ehrlichii*] (WP_116303324.1)	56.84	✓
*PW-xyl11*	383	Endo-1,4-β-xylanase [*Runella* sp. YX9] (WP_114464289.1)	69.94	✓
*PW-xyl12*	383	Endo-1,4-β-xylanase [*Runella* sp. YX9] (WP_114464289.1)	70.45	✓
*PW-xyl13*	390	1,4-β-xylanase [*Flavobacteriia* bacterium] (RUA15923.1)	58.79	✓
*PW-xyl14*	391	Endo-1,4-β-xylanase [*Confluentibacter lentus*] (WP_100611294.1)	71.90	✓
*PW-xyl15*	396	TPA: 1,4-β-xylanase [*Planctomycetes* bacterium] (HAK94543.1)	77.31	✓
*PW-xyl16*	403	1,4-β-xylanase [*Butyrivibrio* sp. NC2007] (WP_022770809.1)	52.21	
*PW-xyl17*	413	TPA: 1,4-β-xylanase [*Cyanobacteria* bacterium UBA11371] (HAZ45611.1)	48.50	✓
*PW-xyl18*	415	Endo-1,4-β-xylanase, GH35 family [*Bacteroidales* bacterium 6E] (GAP68157.1)	58.29	
*PW-xyl19*	420	Endo-1,4-β-xylanase [*Planctomycetaceae* bacterium] (RPJ36220.1)	74.31	
*PW-xyl20*	422	1,4-β-xylanase [*Microbispora* sp. GKU 823] (WP_079313614.1)	63.19	✓
*PW-xyl21*	424	Endo-1,4-β-xylanase [*Bacteroidales* bacterium 6E] (GAP68157.1)	64.34	
*PW-xyl22*	425	Endo-1,4-β-xylanase [*Bacteroidales* bacterium 6E] (GAP68157.1)	64.92	
*PW-xyl23*	426	Endo-1,4-β-xylanase [*Bacteroidales* bacterium 6E] (GAP68157.1)	60.21	
*PW-xyl24*	451	Endo-1,4-β-xylanase Z [*Tannerella forsythia*] (SCQ20945.1)	34.57	
*PW-xyl25*	452	1,4-β-xylanase [*Thermogutta terrifontis*] (WP_095416323.1)	55.15	
*PW-xyl26*	460	1,4-β-xylanase [*Planctomycetes* bacterium B3_Pla] (TKJ36372.1)	60.67	
*PW-xyl27*	475	β-1,4-xylanase [*Mesotoga infera*] (KUK68222.1)	76.09	
*PW-xyl28*	476	Endo-1,4-β-xylanase [*Cyanothece* sp. PCC 7822] (WP_013324907.1)	27.16	
*PW-xyl29*	492	1,4-β-xylanase [*Aphanothece sacrum*] (WP_124970705.1)	27.23	
*PW-xyl30*	495	1,4-β-xylanase [*Chthonomonas calidirosea* T49] (CCW34445.1)	43.40	
*PW-xyl31*	496	Endo-1,4-β-xylanase [*Treponema azotonutricium*] (WP_015711732.1)	69.70	
*PW-xyl32*	502	1,4-β-xylanase [*Bacteroidetes* bacterium GWE2_42_39] (OFY01282.1)	63.91	
*PW-xyl33*	583	Endo-1,4-β-xylanase [*Geitlerinema* sp. PCC 9228] (WP_084639516.1)	50.00	
*PW-xyl34*	778	Endo-1,4-β-xylanase [*Marinilabilia salmonicolor*] (WP_010663012.1)	93.00	
*PW-xyl35*	647	Endo-1,4-β-xylanase [*Hymenobacter gummosus*] (WP_126694067.1)	44.70	
*PW-xyl36*	711	Endo-1,4-β-xylanase, GH35 family [*Bacteroidales* bacterium 6E] (GAP68157.1)	69.05	
*PW-xyl37*	723	Endo-1,4-β-xylanase B [uncultured *Bacteroides* sp.] (SCJ93802.1)	71.01	✓
*PW-xyl38*	724	Endo-1,4-β-xylanase [*Ignavibacteriae* bacterium] (RPI05742.1)	50.14	✓
*PW-xyl39*	746	Endo-1,4-β-xylanase [*Moorea producens*] (WP_083305227.1)	51.95	✓
*PW-xyl40*	746	TPA: 1,4-β-xylanase [*Cyanobacteria* bacterium] (UBA11371] (HAZ45611.1)	57.59	

^a^AA is the length of the amino acid;

^b^If the last column is labeled

✓, the expressed xylanase gene has xylanase activity

The primers were designed and used for the cloning of xylanase genes in the pET28 expression plasmid (Additional file 1: Table S1). The DNA extracted from the microbiota of the targeted PPWT plant was used as template, and Phanta Max Super-Fidelity DNA polymerase (NO. P505, Nanjing, China) was used for gene amplification. All 40 GH10 family genes were cloned and verified by Sanger sequencing; their accession numbers are MW124392–MW124431 in the GenBank database.

The genes were separately ligated into pET28a (+) using the homologous recombinant enzyme (Novogene, Beijing, China) according to the manufacturer’s instructions. Subsequently, the recombinant mixtures were transformed into the *E. coli* TOP10 strain. Following verification, the recombinant plasmids obtained from the TOP10 strain were further transformed into the *E. coli* BL21 strain.

### Expression and detection of recombinant proteins

The *E. coli* BL21 strains including the recombinant plasmids were inoculated into 100 mL LB medium containing kanamycin (50 µg/mL) in a 500 mL shake flask, and incubated at 37 ℃. Once each *E. coli* BL21 culture harboring a xylanase gene had grown to reach an OD600 value of 0.8, 200 mM IPTG was added to the culture in order to induce xylanase expression. After cultivation for further 16 h at 20 ℃, cells were harvested by centrifugation at 12,000 rpm for 5 min. Harvested cells were washed with phosphate buffered saline (PBS) buffer (pH 7.4), and then disrupted with supersonic waves (Xiaomei, Kunshan, China) on ice for 15 min using the method of 5 s pulses and 10 s intervals (Yan et al. 2013). Finally, cells were centrifuged at 12,000 rpm for 10 min, and the supernatant was used for the xylanase activity assay.

The DNS method was applied to determine xylanase activity based on the release of reducing sugar from the mixture of xylanase and beechwood substrate (Wei et al. 2015). A cell supernatant of 25 µL volume was mixed with 25 µL xylan (4 mg/mL), and the mixture was left to react for an hour in total, including 20 ℃ for 20 min, 40 ℃ for 20 min, and 60 ℃ for further 20 min. Subsequently, 50 µL DNS reagent was added to tubes to stop the enzymatic reaction, and the mixtures were further incubated in a 95 ℃ water bath for 5 min. Finally, the crude activity of xylanase could be determined (Yan et al. 2013).

### Recombinant protein expression and purification

Two BL21 strains with xylanase genes of *PW-xyl9* or *PW-xyl37* were selected for further characterization. The corresponding strains were incubated overnight in a shaker at 37 ℃ and 200 rpm; 4 mL of the overnight culture was then inoculated to 200 mL fresh LB medium in a 1 L shake flask for further culturing. Once the OD600 value of the two strains had reached 0.8, 200 mM IPTG was added, and the culture was cultivated at 20 ℃ for an additional 16 h. Cells were harvested and washed 3 × with PBS buffer, and were suspended in 20 mL PBS buffer with 1 mM phenylmethylsulfonyl fluoride (PMSF) which was protease inhibitor. The cells were disrupted by sonication for a total length of 15 min (Xiaomei, Kunshan, China), with setting parameters of 150 W, 3 s on, and 5 s off. The cell suspensions were centrifuged at 12,000 rpm at 4 ℃ for 20 min, and the supernatant was collected as crude enzyme. Magnetic beads (PuriMag, Xiamen, China) were used for protein purification, and the purified proteins obtained were concentrated in 10 kDa ultrafiltration tubes (Merck, Millipore, USA). The purity of proteins was determined by sodium dodecyl sulfate–polyacrylamide gel electrophoresis (SDS-PAGE), and their concentrations were measured with the Bradford method (Sangon Biotech, Shanghai, China).

### Enzymatic assay

Enzyme activity was measured in 1% beechwood xylan dissolved in PBS Buffer with a pH value of 7.4. The enzyme reaction mixtures comprised of 50 µL 2% xylan and 50 µL enzyme solution. Mixtures were left to react at 50 ℃ for 10 min, and the reaction was terminated with the addition of 100 µL DNS reagent solution. The resulting reaction mixtures of 200 µL volume were further incubated at 95 ℃ for 5 min. The final solutions were cooled on ice, and 120 µL of each reaction mixture was used for absorbance measurement at 540 nm (Teixeira et al. 2012). The specific enzyme activity (U/mg protein) was defined as the amount of xylose released/min/mg enzyme. All data in the current study are presented as the means of triplicate experiments.

The optimum pH of xylanases was determined in buffers with different pH values. The sodium citrate–phosphate buffer was used from pH 4.5–6, Tris–HCl buffer was applied from pH 6–9, and glycine–NaOH buffer was used from pH 9–10. In order to determine the optimal pH and temperature for each enzyme, xylanase reactions were carried out at 50 ℃ and pH = 7, respectively. The highest xylanase activity detected in these experiments was defined as 100%.

### Analyses of kinetic parameters and xylanase characterization

The kinetic parameters of K_m_ and V_max_ were determined. The reaction was carried out for 3 min at the optimum temperature and pH determined for each xylanase. Values of V_max_ and K_m_ were calculated based on the data obtained. The Prosite protein database (https://prosite.expasy.org/) was used to predict the structural domains and active sites of PW-xyl9 and PW-xyl37. The structural domains and signal peptides of proteins were predicted using the SignalP-5.0 server (Almagro Armenteros et al. 2019). Homologous modeling was conducted for the amino acid sequences of PW-xyl9 and PW-xyl37 with the SWISS-MODEL (Arnold et al. 2006). The quality of structural models was evaluated through the SAVES V5.0 server, and results were visualized by the PyMol molecular visualization system.

## Results

### Expression and analysis of the 40 xylanase genes

**Fig. 1:**
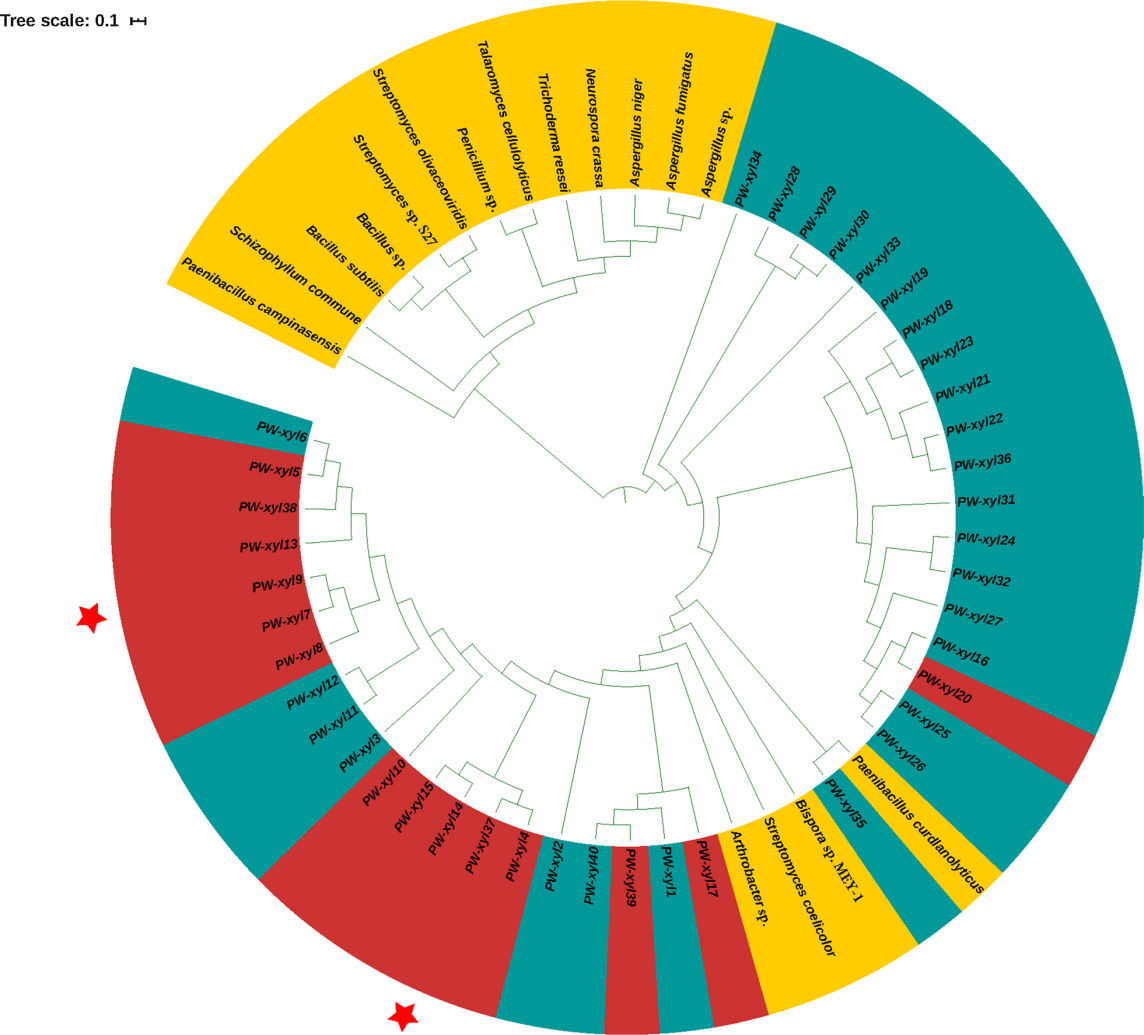
The phylogenetic tree of the 40 verified xylanase genes and some xylanase genes downloaded from available database based on amino acid identity analyses with MEGA. The genes labeled yellow are xylanases with high activity retrieved from the enzyme repository of the ExPaSy database accessed at https://enzyme.expasy.org/. The microbial gene sources and their accession numbers are: *Aspergillus niger* XP_001388522.1, *Bispora* sp. *MEY-1* ACS96449.1, *Penicillium* sp. ACY70400.1, *Schizophyllum commune* P35809.1, *Talaromyces cellulolyticus* BAO51921.1, *Trichoderma reesei* ACB38137.1, *Aspergillus* sp. Q4WG11.1, *Neurospora crassa* Q7SDQ1, *Streptomyces coelicolor* NP_733679.1, *Bacillus subtilis* P18429, *Streptomyces* sp. *S27* ACF57948.1, *Paenibacillus campinasensis* Q2I6W5, *Paenibacillus curdlanolyticus* BAK22544.1, *Bacillus* sp. ACR47980.1, *Arthrobacter* sp. AGC01501.1, *Streptomyces olivaceoviridis* AHK22787.1. The 40 gene sequences with blue and red markings were expressed in *E. coli*; the ones marked red were expressed with xylanase activity, whereas the ones marked blue were expressed without xylanase activity. The two proteins labeled with two red star pentagons were selected to determine their xylanase properties after purification

Using the primers designed with previous metagenomic data, 40 xylanase genes were cloned. These 40 xylanase genes were assigned to the GH10 family of glycoside hydrolases. These genes showed 27.16–98.67% identity with genes in the GenBank database. Among them, the *PW-Xyl9* gene had 89.3% identity with an endo-1,4-β-xylanase gene from *Bacteroidales*, while the *PW-Xyl28* gene showed 27.16% identity with an endo-1,4-β-xylanase of *Cyanothece* sp*.* PCC 7822. All the 40 cloned xylanase genes were different from xylanases with high activities in the BRENDA database (https://www.brenda-enzymes.org/) (Fig. 1). Fourteen of all 40 xylanase genes expressed in *E. coli* presented xylanase activity (Fig. 1 and Table 1).

### Expression of *PW-xyl9* and* PW-xyl37*

Among the 14 active xylanases, 11 crude xylanases had high xylanase activities (Additional file 1: Figure S1 and Table 1). The crude enzymes of PW-xyl9 and PW-xyl37 showed higher xylanase activities compared with the remaining 12 active xylanases. Therefore, PW-xyl9 and PW-xyl37 were selected to determine their xylanase properties. These two proteins were subsequently expressed in *E. coli* and successfully purified (Additional file 1: Figure S2). Their molecular weights were determined as about 43 and 82 kDa, respectively, which were consistent with the predicted molecular weight based on the two corresponding genes (Table 1). According to our prediction, the active sites of PW-xyl9 are GLU162 and GLU267, whereas the active sites of PW-xyl37 are GLU 166 and GLU282, respectively **(**Additional file 1: Figure S3**)**. Moreover, both PW-xyl9 and PW-xyl37 have signal peptides, indicating that these two xylanases may be extracellular xylanases which could be secreted and function outside of cells in the natural environment (Ohta et al. 2011).

### Enzymatic characteristics of PW-xyl9 and PW-xyl37

**Fig. 2:**
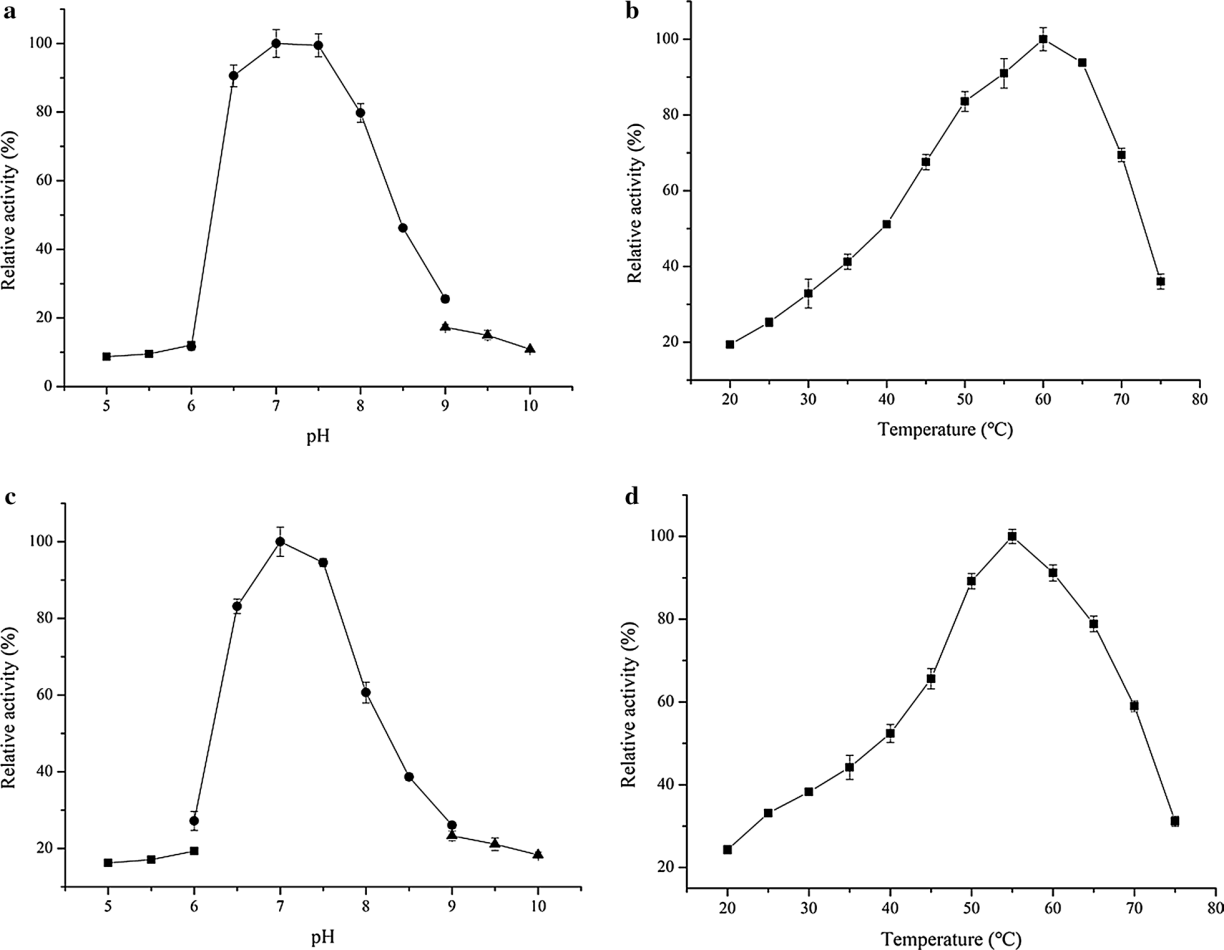
Enzymatic characteristics of PW-xyl9 and PW-xyl37. **a** Optimal pH value for PW-xyl9. **b** Optimal temperature value for PW-xyl9. **c** Optimal pH value for PW-xyl37. **d** Optimal temperature value for PW-xyl37. The pH range was 5–10, and the buffer systems used were: citrate–phosphate (■) for the pH 5–6 range, Tris–HCl (●) for the pH 6–9 range, glycine–NaOH (▲) for the pH 9–10 range. The value represents the mean value of triplicate experiments, and the error bar indicates standard deviation

The optimal pH and temperature of PW-xyl9 were determined as pH 7 and 60 ℃, respectively; while the optimal pH and temperature of PW-xyl37 were pH 7 and 55 ℃, respectively (Fig. 2). It showed that the xylanase activity of PW-xyl9 can keep > 80% of its highest value from pH 6.5 to pH 8.0, and keep > 50% of its highest value from 40 to 70 ℃. With respect to PW-xyl37, its xylanase activity could maintain > 80% of its highest value pH 6.5 to pH 7.5, whereas its xylanase activity could maintain > 50% of its highest value from 40 to 70 ℃.

Considering the optimal condition of beechwood as substrate, the specific xylanase activities of PW-xyl9 and PW-xyl37 were 438.4 U/mg protein and 198.1 U/mg protein, respectively. With regards to enzymatic kinetics, PW-xyl9 had a *K*_m_ value of 5.95 g/L, *k*_*cat*_ of 59.3 S^−1^, Vmax was 0.11 µM/min, and catalytic efficiency (Kcat/K_M_) of 9.97 (Additional file 1: Figure S4a and Table 2)_._ The relevant values for PW-xyl37 were *K*_m_ = 4.65 g/L, *k*_*cat*_ = 36.1 S^−1^, V_max_ = 0.125 µM/min, and K_cat_/K_M_ = 7.76, respectively (Additional file 1: Figure S4b and Table 2)_._

**Table 2:** Enzyme activity parameters of PW-Xyl9 and PW-Xyl37

Names	K_m_ (mg/ml)	V_max_ (µM/min)	K_cat_ (sec^−1^)	K_cat_/K_m_ (mL*mg^−1^*sec^−1^)
PW-Xyl9	5.95	0.110	59.3	9.97
PW-Xyl37	4.65	0.125	36.1	7.76

### Bioinformatics analysis of PW-xyl9 and PW-xyl37

The SWISS-MODEL method was used to predict the quaternary structures of PW-xyl9 and PW-xyl37. The identity of amino acid sequence between PW-xyl9 and the xylanase (PDB: 1uqy) of *Cellvibrio mixtus* was 43% (Pell et al. 2004). This xylanase (PDB: 1uqy) was selected as template to construct the 3D structure model of PW-xyl9. The expected accuracy of the model for PW-xyl9 was GMQE = 0.73, with a QMEAN value of −0.45. The identity of amino acid sequence between PW-xyl37 and the xylanase (PDB: 5AY7) derived from goat rumen microbiota was 51%, and this structure was selected as template to construct a 3D structure model of PW-xyl37. The GMQE and QMEAN values of PW-xyl37 were determined as 0.38 and −1.39, respectively (Additional file 1: Figure S5). These data indicate that the two predicted models are a good match with their template proteins (Benkert et al. 2009).

The quality of the built structure models was confirmed using the SAVES V5.0 server. The VERIFY value, ERRAT value, and the value of residues in most favored regions of PW-xyl9 were shown as 91.01, > 95.2096, and 92.5%, respectively (Additional file 1: Figure S5). Meanwhile, the VERIFY value, ERRAT value, and value of residues in most favored regions of PW-xyl37 was 92.56, > 96.5839, and 90.1, respectively (Additional file 1: Figure S5). Since a VERIFY value of > 80%, an ERRAT value of > 85%, and value of residues in most favored regions of > 90% are indicators of a good quality of a predicted model, the structural models predicted herein are good representatives of the real structures of PW-xyl9 and PW-xyl37.

**Fig. 3:**
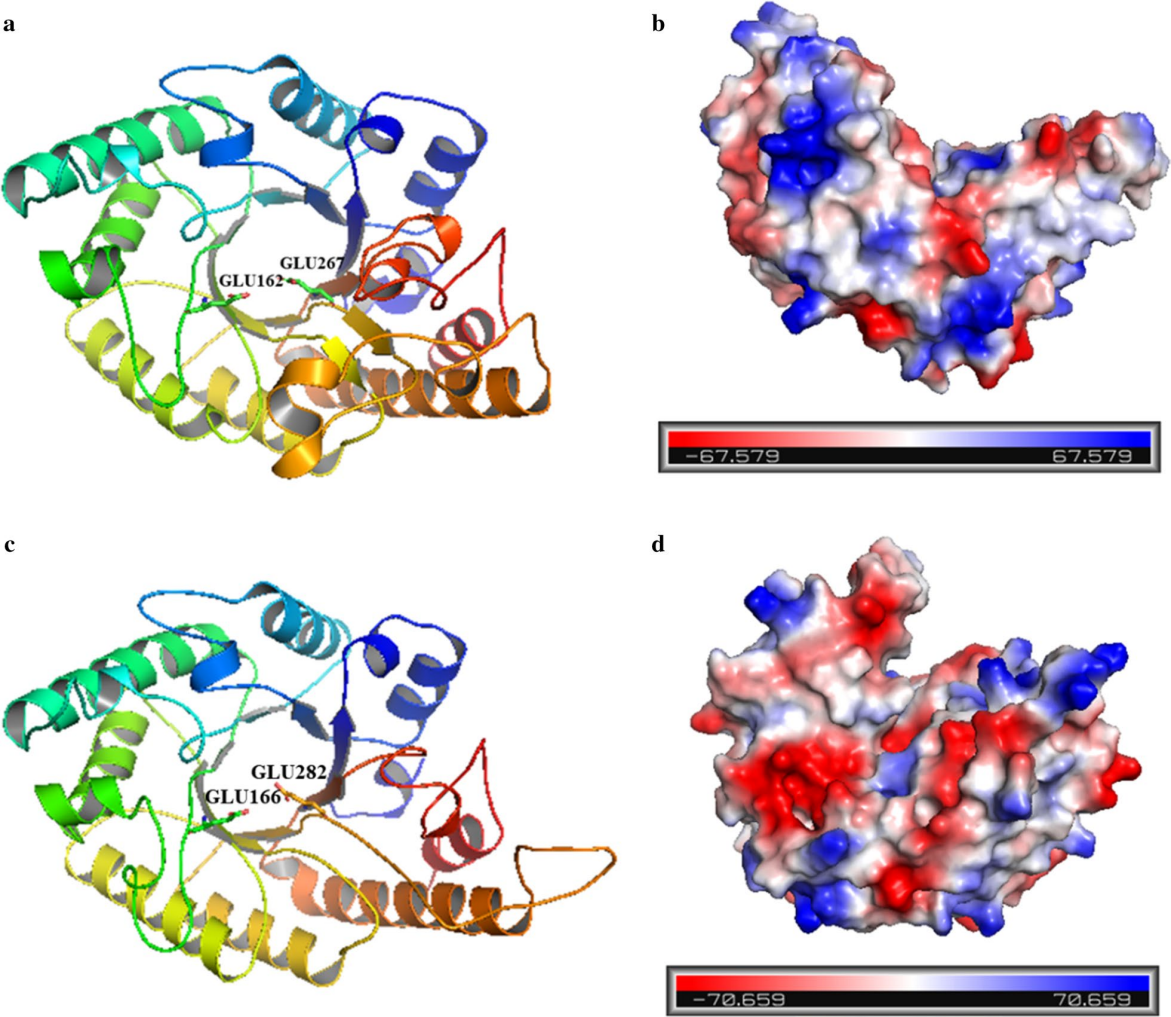
3D model and electrostatic properties of PW-Xyl9 and PW-Xyl37. **a** 3D model of PW-Xyl9. GLU162 and GLU267 are the active sites of PW-Xyl9. **b** Electrostatic properties of PW-Xyl9. **c** 3D model of PW-Xyl37. GLU166 and GLU282 are the active sites of PW-Xyl37. **d** Electrostatic properties of PW-Xyl37. Enzyme electrostatic properties were predicted with PyMOL software. The red surface indicates that the relevant area has strong positive electrostatic properties, while the blue surface indicates the area has strong negative electrostatic properties

The predicted 3D models of PW-xyl9 and PW-xyl37 demonstrate that they harbor a classic (β/α)_8_-barrel fold (Fig. 3), which is present in about 10% of known enzyme structures and is the characteristics of xylanases of the GH10 family, further evidencing that PW-xyl9 and PW-xyl37 are indeed GH family xylanases (Sterner and Hocker 2005). Based on analysis by APBS software, PW-xyl9 and PW-xyl37 are electronegative (Fig. 3).

## Discussion

With the improvement of omics technologies and microbiome analysis, many efficient lignocellulose degradation systems have been investigated (Liu et al. 2019; Stewart et al. 2018; Wei et al. 2015). Diverse lignocellulase genes have been recovered from natural lignocellulose-degrading microbiota, including the termite hindgut and the cow rumen (Al-Darkazali et al. 2017; Joshi et al. 2020; Verma et al. 2013). About one million GH gene modules have been identified and included in the CAZy database (Lombard et al. 2014). Moreover, large amounts of novel lignocellulase genes from a particular PPWT microbiota were identified in our previous study (Liang et al. 2021). As xylan is one of the most abundant components of biomass in nature, it is of great importance to screen novel efficient xylanases from natural microbiota (Madeira et al. 2017). Compared with a lab-scale biogas digester, a diverse array of xylanase genes from the GH10 family and a number of xylanase genes from the GH11 family were identified in the abovementioned PPWT microbiota, showing that GH10 family xylanases in such microbiota might perform well in xylan degradation (Liang et al. 2021). Two xylanases with high activities were characterized in the present study, suggesting that GH10 family xylanases might play essential roles in xylan degradation by PPWT microbiota (Liang et al. 2021).

The optimal pH of the two characterized xylanases was pH 7. Considering that the functional pH for the studied microbiota was in the range of pH 7–9 (Fig. 2), xylanases and their hosts occurring in the microbiota might show adaptation to the pH environment of PPWT. Optimal temperatures for these two characterized xylanases were 55 and 60 ℃, respectively, however, they exhibited high activities from 40 to 75 ℃. The running temperature of the studied PPWT microbiota was 38 ℃ (Liang et al. 2021), suggesting that these xylanases show robustness. In fact, the types of lignocellulase identified in mesophilic biogas digesters and the termite hindgut exhibited high activities at temperatures above 40 ℃ (Qian et al. 2015; Yan et al. 2013), which is consistent with the results of our study. The two xylanases characterized herein have signal peptides (Roslan et al. 2020), indicating that they might be secreted and functional extracellularly in PPWT bioreactors. The xylanase activities in the PPWT bioreactors were low, which might be explained by the suboptimal running temperature and complex environment in these bioreactors (Liang et al. 2021). Furthermore, a complex mixture of substrates was present in the pulp and paper wastewater, and the xylan content might not be high, thus even low xylanase activity in the bioreactors was sufficient to degrade all the xylan in the wastewater.

Xylanases are mainly used in the pulp bleaching, baking, and food industries (Walia et al. 2017). Some of these technologies require xylanases to be active at higher temperatures and alkaline environments (Mhiri et al. 2020). The two xylanases characterized in this study functioned at conditions of a wide range of pH and higher temperature, evidencing their potential application in the pulp bleaching and other industries. Commercial xylanases are required to be thermostable and robust to recalcitrant conditions. Analyses of surface electrostatic potential indicate that PW-xyl9 and PW-xyl37 are electronegative, suggesting that these two xylanases might have good thermal stability (Zhou et al. 2019). Compared with the xylanases deposited in the BRENDA database, the activities of the two characterized xylanases are not high, and further engineering of these two enzymes for higher xylanase activities and higher thermostability are necessary. Our enzyme structure models identified the active sites of the two characterized xylanases, which might be useful for the future engineering of xylanases with enhanced activity and thermal stability. The directed evolution and rational design guided strategies can be used to improve the characters of these two enzymes (Xing et al. 2021).

Environmental biotechnology has recovered large amounts of functional genes from natural microbiota, however, most genes have not been characterized to date (Ariaeenejad et al. 2019; Stewart et al. 2018). Some of the xylanase genes identified from PPWT microbiota in this paper showed > 60% identities with known genes in the GenBank database (Table 1), while the function of these genes was not verified against GenBank. Herein, 14 genes with xylanase activities were identified by expressing a total of 40 xylanase genes in *E. coli*, showing that the heterologous expression system of xylanase genes in *E. coli* cannot express all recovered genes. Therefore, the future development of novel efficient microbial chassis systems for functional environmental gene expression might improve the understanding of functional genes and their respective catalytic mechanisms in natural microbiota (de Paula et al. 2019; Kim et al. 2020). Nowadays, the progressive engineering of expression systems in microbial hosts, such as *Saccharomyces cerevisiae*, might lead to an efficient microbial cell factory for lignocellulose degradation (Tang et al. 2017).

In the present study, we cloned and expressed 40 xylanase genes, recovered from a particular PPWT microbiota, in *E. coli*. Fourteen of these genes showed xylanase activity, and two with high xylanase activity were characterized. Moreover, they function under a wide range of pH and temperature conditions, indicating they have potential application in industry. Bioinformatics analyses were used to give an insight into the structure of these two characterized xylanases, which might be applicable for the improvement of thermostability and other properties of industrial xylanases through protein engineering in the future.

## Supplementary information

**Additional file 1.** Additional Table and Figures.

## Data Availability

The accession numbers of the 40 xylanase genes are MW124392–MW124431 in GenBank database.
