# Supplementary material for: Characterization of efficient xylanases from industrial-scale pulp and paper wastewater treatment microbiota
Source: AMB Express. 2021 Jan 19;11:19. doi: 10.1186/s13568-020-01178-1 (PMC7815853; doi:10.1186/s13568-020-01178-1)
Supplement: Supplementary file 1 — Additional file 1. Additional Table and Figures. [file 13568_2020_1178_MOESM1_ESM.pdf]

AMB Express

Supplementary Information

Characterization of efficient xylanases from industrial-scale pulp and paper wastewater treatment microbiota

Jia Wang[1], Jiawei Liang[2], Yonghong Li[1*], Lingmin Tian[3*], Yongjun Wei[1*]

[1] Key Laboratory of Advanced Drug Preparation Technologies, Ministry of Education, School of Pharmaceutical Sciences, Zhengzhou University, Zhengzhou, PR China

[2] College of Public Health, Zhengzhou University, Zhengzhou, Henan 450001, PR China

[3] Department of Food Science and Engineering, Jinan University, Guangzhou 510632, PR China

Corresponding authors

Yonghong Li

Email: lyh224@163.com

Phone number: 0086-37167781908

Lingmin Tian

Email: tianlinmin@163.com

Phone number: 0086-18620796345

Yongjun Wei

Email: yongjunwei@zzu.edu.cn

Phone number: 0086-37167781908

Supplementary Table S1 Primers used in this study.

Gene names	primer names	Primer sequences (from 5’ to 3’)
PW-xyl1	PW-xyl1_F	GCCTGGTGCCGCGCGGCAGCATGGATACAACCCATATCGCATCTCTAC
	PW-xyl1_R	GGTGCTCGAGTGCGGCCGCGGCAGCCTGCAGCGC
PW-xyl2	PW-xyl2_F	GCCTGGTGCCGCGCGGCAGCATGAGCGAAGTTCTTTCTCTGGCAAAAAG
	PW-xyl2_R	GGTGCTCGAGTGCGGCCGCCCGCCCGGCGGCGTCAATG
PW-xyl3	PW-xyl3_F	GCCTGGTGCCGCGCGGCAGCATGAAGAAGAGTATTCTCATGTTGTCGG
	PW-xyl3_R	GGTGCTCGAGTGCGGCCGCATTAAATAAGTCTATAATTTTATTTATAACCGG
PW-xyl4	PW-xyl4_F	GCCTGGTGCCGCGCGGCAGCATGAAAACCAGATTTAAAATTAATCAATGC
	PW-xyl4_R	GGTGCTCGAGTGCGGCCGCGAATTTCACGACCTCCCAGAAC
PW-xyl5	PW-xyl5_F	GCCTGGTGCCGCGCGGCAGCATGAAGAAGACAGTCCCCATTGCAG
	PW-xyl5_R	GGTGCTCGAGTGCGGCCGCTTGTCGCTGGGCGGCCTTC
PW-xyl6	PW-xyl6_F	GCCTGGTGCCGCGCGGCAGCGTGAGTAAGCAGAAGATTGTCACCG
	PW-xyl6_R	GGTGCTCGAGTGCGGCCGCTTGCTGCCGCGCGGCTTTCAC
PW-xyl7	PW-xyl7_F	GCCTGGTGCCGCGCGGCAGCATGAGCAAGTTGAGAAATTTTCTGATGGC
	PW-xyl7_R	GGTGCTCGAGTGCGGCCGCCTTTTTAGTGTACTTATCCAAAAACGGC
PW-xyl8	PW-xyl8_F	GCCTGGTGCCGCGCGGCAGCATGAAAATCAAGACCATTACCGCAATAATTG
	PW-xyl8_R	GGTGCTCGAGTGCGGCCGCTCTTTTTCGCTTGTATTTTTTTGCC
PW-xyl9	PW-xyl9_F	GCCTGGTGCCGCGCGGCAGCATGAAATTGAAATCGTATTATTTTGTAATTTC
	PW-xyl9_R	GGTGCTCGAGTGCGGCCGCTTGGTTTGCTTCTTTGATTATTG
PW-xyl10	PW-xyl10_F	GCCTGGTGCCGCGCGGCAGCATGCCGATGTCACGCCGCCG
	PW-xyl10_R	GGTGCTCGAGTGCGGCCGCGCCGGAGTTCTTCAGCGCG
PW-xyl11	PW-xyl11_F	GCCTGGTGCCGCGCGGCAGCATGCATAGGATCGGACTGCTGCTG
	PW-xyl11_R	GGTGCTCGAGTGCGGCCGCCCGGTTCCTAGAGGGGAGCTG
PW-xyl12	PW-xyl12_F	GCCTGGTGCCGCGCGGCAGCATGCACAAAACCGGCCTGCTG
	PW-xyl12_R	GGTGCTCGAGTGCGGCCGCCGGGCGAGTGAAGGGGATC
PW-xyl13	PW-xyl13_F	GCCTGGTGCCGCGCGGCAGCATGATTAAATTAGGATGTATCAAAGGTC
	PW-xyl13_R	GGTGCTCGAGTGCGGCCGCTTTATTCATAAGGTCTATCACACTTAAATATG
PW-xyl14	PW-xyl14_F	GCCTGGTGCCGCGCGGCAGCATGATTGACAAAAAACTGATTACCAGC
	PW-xyl14_R	GGTGCTCGAGTGCGGCCGCTTTATAGGAACTTGCTTCAATAACGG
PW-xyl15	PW-xyl15_F	GCCTGGTGCCGCGCGGCAGCATGAAATGCTGGATGCTAGGCCTCG
	PW-xyl15_R	GGTGCTCGAGTGCGGCCGCTTGTGGCGCCGTCGGGAC
PW-xyl16	PW-xyl16_F	GCCTGGTGCCGCGCGGCAGCGTGAGCACGGCAGAGACGATC
	PW-xyl16_R	GGTGCTCGAGTGCGGCCGCCAGAGCAAGCGCGAGCGCAG
PW-xyl17	PW-xyl17_F	GCCTGGTGCCGCGCGGCAGCATGCACAAAAGAACTCACCAGGCG
	PW-xyl17_R	GGTGCTCGAGTGCGGCCGCTGGATTTTGAAGTAAATCCATTATGGCG
PW-xyl18	PW-xyl18_F	GCCTGGTGCCGCGCGGCAGCATGCGCATTTACGGCGCTCTGTTC
	PW-xyl18_R	GGTGCTCGAGTGCGGCCGCCGGACGCGCCACAGAGAC
PW-xyl19	PW-xyl19_F	GCCTGGTGCCGCGCGGCAGCATGCAGCAATACAGCAGGGTCTCAG
	PW-xyl19_R	GGTGCTCGAGTGCGGCCGCGTTGCCTAGTGTCACGACCTG
PW-xyl20	PW-xyl20_F	GCCTGGTGCCGCGCGGCAGCATGACCGACCATTCCGCGTACC
	PW-xyl20_R	GGTGCTCGAGTGCGGCCGCATCAAAATGTACCTCGACCGAAAG
PW-xyl21	PW-xyl21_F	GCCTGGTGCCGCGCGGCAGCATGAACTACCTGCCAGCTGAAGC
	PW-xyl21_R	GGTGCTCGAGTGCGGCCGCTCTTAAATCAACCACAACTTTTCCTTC
PW-xyl22	PW-xyl22_F	GCCTGGTGCCGCGCGGCAGCATGAAAAATTCCGTTTATTCAGCTAAAG
	PW-xyl22_R	GGTGCTCGAGTGCGGCCGCCTTACTCCTGAAATCAACAATTACG

PW-xyl23	PW-xyl23_F	GCCTGGTGCCGCGCGGCAGCATGAGAACGCGTGGAAGAACG
	PW-xyl23_R	GGTGCTCGAGTGCGGCCGCTTCCGATTTCAGTGAGACCACCTTC
PW-xyl24	PW-xyl24_F	GCCTGGTGCCGCGCGGCAGCATGAGCCGACAACCAGTCGCG
	PW-xyl24_R	GGTGCTCGAGTGCGGCCGCACGAGCGTCCAACTCGAGTTTG
PW-xyl25	PW-xyl25_F	GCCTGGTGCCGCGCGGCAGCATGAAGATTCTCTGTTCTGCGATCC
	PW-xyl25_R	GGTGCTCGAGTGCGGCCGCCCTCGACAGACGGACCTCG
PW-xyl26	PW-xyl26_F	GCCTGGTGCCGCGCGGCAGCATGGCAAAGCGAAACTCAGGATGCG
	PW-xyl26_R	GGTGCTCGAGTGCGGCCGCCCGGTCAGCGAGCGATTCCG
PW-xyl27	PW-xyl27_F	GCCTGGTGCCGCGCGGCAGCATGTCGTTTGAGCAGTCTTCTTCGAAG
	PW-xyl27_R	GGTGCTCGAGTGCGGCCGCTCGCACCTCCTGTTCCTTTCCG
PW-xyl28	PW-xyl28_F	GCCTGGTGCCGCGCGGCAGCATGAGGCAAACTGTAACTATACGAGCAT
	PW-xyl28_R	GGTGCTCGAGTGCGGCCGCATCAGGCAGCACACATGATACAAGT
PW-xyl29	PW-xyl29_F	GCCTGGTGCCGCGCGGCAGCGTGAAGTTTCAGGTTTTCAGAGACG
	PW-xyl29_R	GGTGCTCGAGTGCGGCCGCTCGCCTGAGAACCGATAAGTGC
PW-xyl30	PW-xyl30_F	GCCTGGTGCCGCGCGGCAGCTTGAAATTTCTTGTTTATAAAGATGGTCAG
	PW-xyl30_R	GGTGCTCGAGTGCGGCCGCCTGTATATGCTTCAATATCGCTTTC
PW-xyl31	PW-xyl31_F	GCCTGGTGCCGCGCGGCAGCATGAAATGGATCAAGGTGCGCCTTG
	PW-xyl31_R	GGTGCTCGAGTGCGGCCGCTTTTTTGATGATCCGCTCCTTTAGGG
PW-xyl32	PW-xyl32_F	GCCTGGTGCCGCGCGGCAGCATGAGACACACAGTGATGGGATGG
	PW-xyl32_R	GGTGCTCGAGTGCGGCCGCAATGCCGTCGCCGTCTTTCG
PW-xyl33	PW-xyl33_F	GCCTGGTGCCGCGCGGCAGCATGAGCAAAAAATTCATATTAATCGGATCC
	PW-xyl33_R	GGTGCTCGAGTGCGGCCGCCTTTTTAGAGTTCCATAAAGCCTGC
PW-xyl34	PW-xyl34_F	GCCTGGTGCCGCGCGGCAGCATGAAACTGAAATTTCTATTACTTGCTTTAGC
	PW-xyl35_R	GGTGCTCGAGTGCGGCCGCGCGTTTCAGGTACTTTTTGCC
PW-xyl35	PW-xyl35_F	GCCTGGTGCCGCGCGGCAGCATGAAACAAAAAGTGTCACTGTTTTTATTTC
	PW-xyl35_R	GGTGCTCGAGTGCGGCCGCCCTTCCCAGCACCATCATTTTC
PW-xyl36	PW-xyl36_F	GCCTGGTGCCGCGCGGCAGCATGGCCGGAAGCCACATTAATACATTC
	PW-xyl36_R	GGTGCTCGAGTGCGGCCGCCTTCCTTTTATTTGCAAAATCGACTATG
PW-xyl37	PW-xyl37_F	GCCTGGTGCCGCGCGGCAGCATGAAAAAAGCAACACTAATCATTGGTCT
	PW-xyl37_R	GGTGCTCGAGTGCGGCCGCCTTGAACAGCAGTGGAGCCATCTC
PW-xyl38	PW-xyl38_F	GCCTGGTGCCGCGCGGCAGCATGAAAGAAAGACATAGTGCAACG
	PW-xyl38_R	GGTGCTCGAGTGCGGCCGCATTTTTATCCTGAACAACTTTAATCACAGC
PW-xyl39	PW-xyl39_F	GCCTGGTGCCGCGCGGCAGCATGAAGCTCGACAAAAGAATTTTG
	PW-xyl39_R	GGTGCTCGAGTGCGGCCGCTGGTGACTGAGGCCGCGCC
PW-xyl40	PW-xyl40_F	GCCTGGTGCCGCGCGGCAGCATGAAATCAGTCAGAGCTGTGGTCACC
	PW-xyl40_R	GGTGCTCGAGTGCGGCCGCTCGAGCAGCAGCGCCCGTTG
PET28	PET28a_F	GCGGCCGCACTCGAGCAC
	PET28a_R	GCTGCCGCGCGGCACCAGG

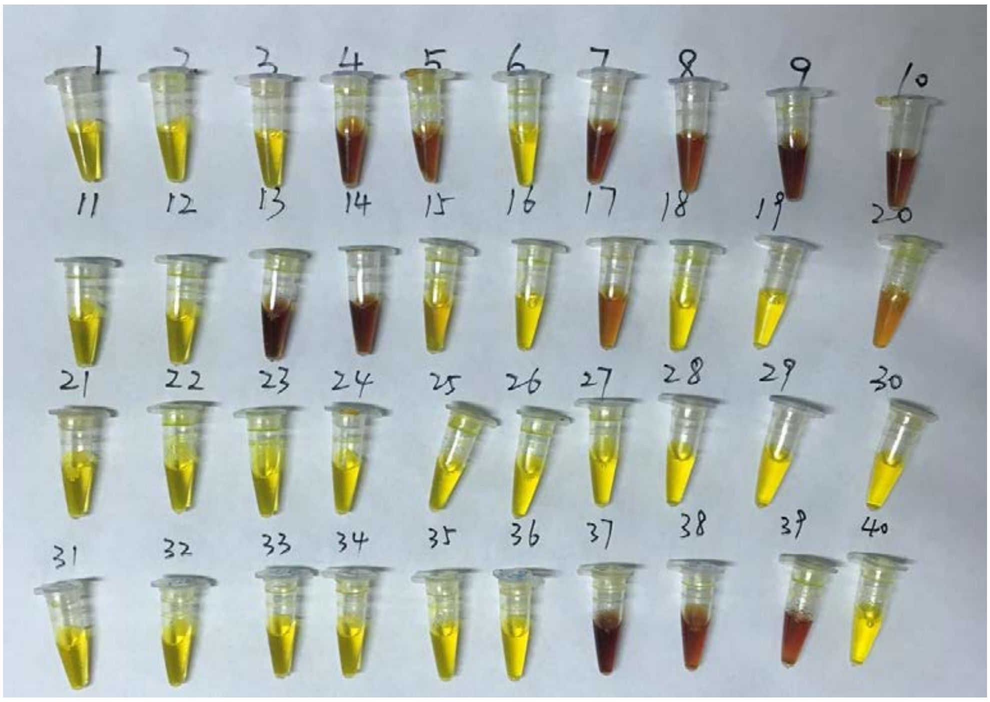

Supplementary Fig. S1 The expression of xylanase genes in *E. coli* strains and their crude enzyme activities. The tubes in red indicate strains harboring xylanase genes have xylanase activity. Numbers of 1-40 are corresponding to the strains harboring genes from *PW-xyl1* to *PW-xyl40*.

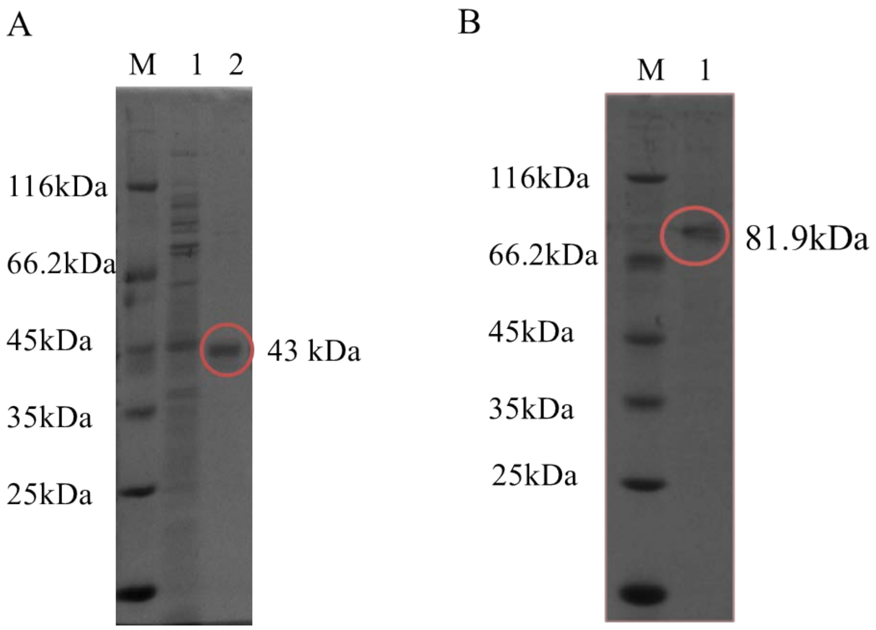

Supplementary Fig. S2 Purification results of the xylanases of PW-xyl9 and PW-xyl37. (A) SDS-PAGE of PW-xyl9. Lanes: M, protein marker; 1, unpurified PW-xyl9; 2, purified PW-xyl9. (B) SDS-PAGE of PW-xyl37. Lanes: M, protein marker; 1, PW-xyl37.

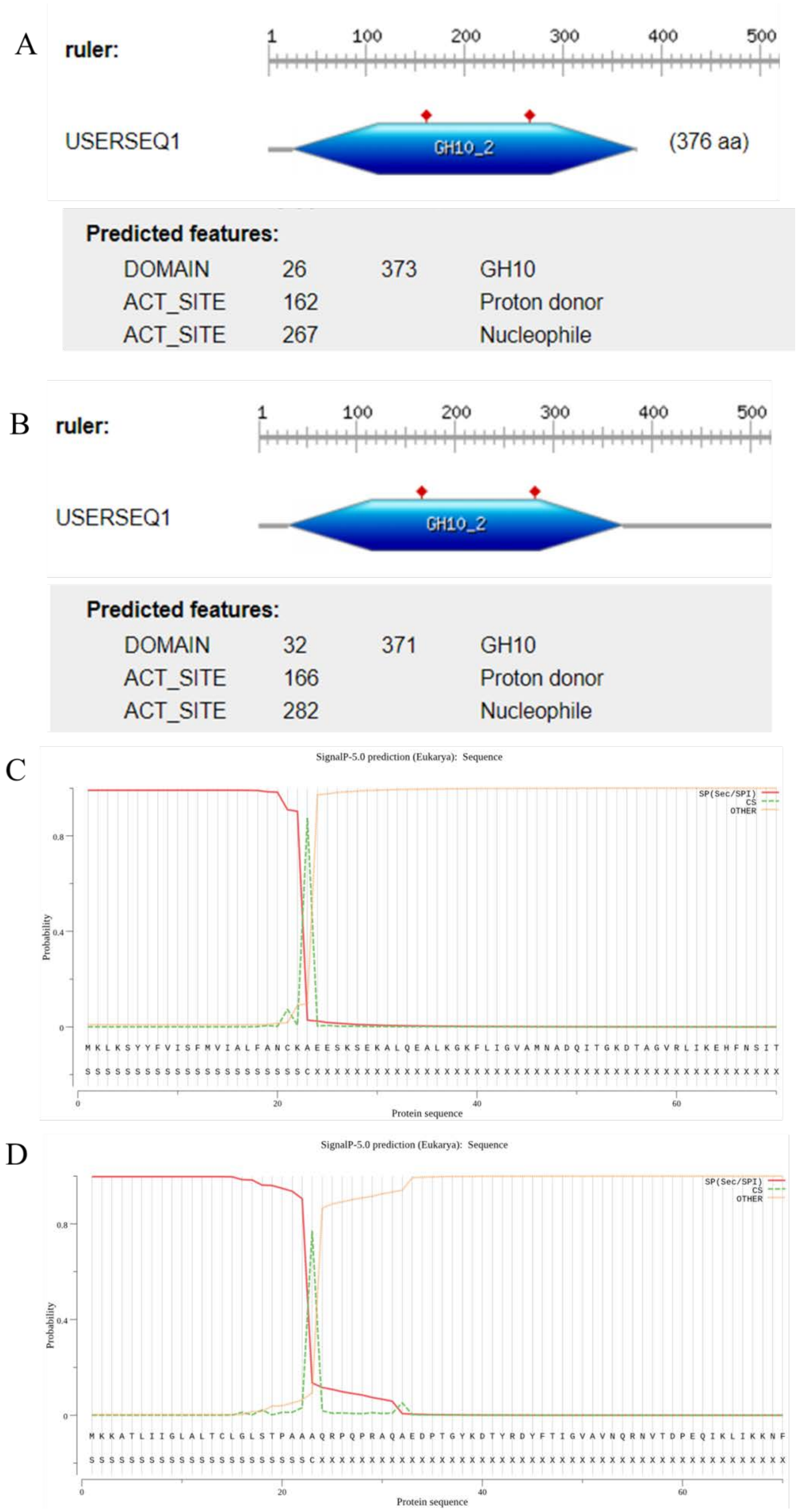

Supplementary Fig. S3 Gene domains and signal peptide analysis of PW-xyl9 and PW-xyl37. (A) Domains and active sites of PW-xyl9 were analyzed with prosite (https://prosite.expasy.org/). (B) Domains and active sites of PW-xyl37 were analyzed with prosite. (C) Signal peptide of PW-xyl9 predicted with SignalP-5 (http://www.cbs.dtu.dk/services/SignalP/). (D) Signal peptide of PW-xyl37 predicted with SignalP-5.

A
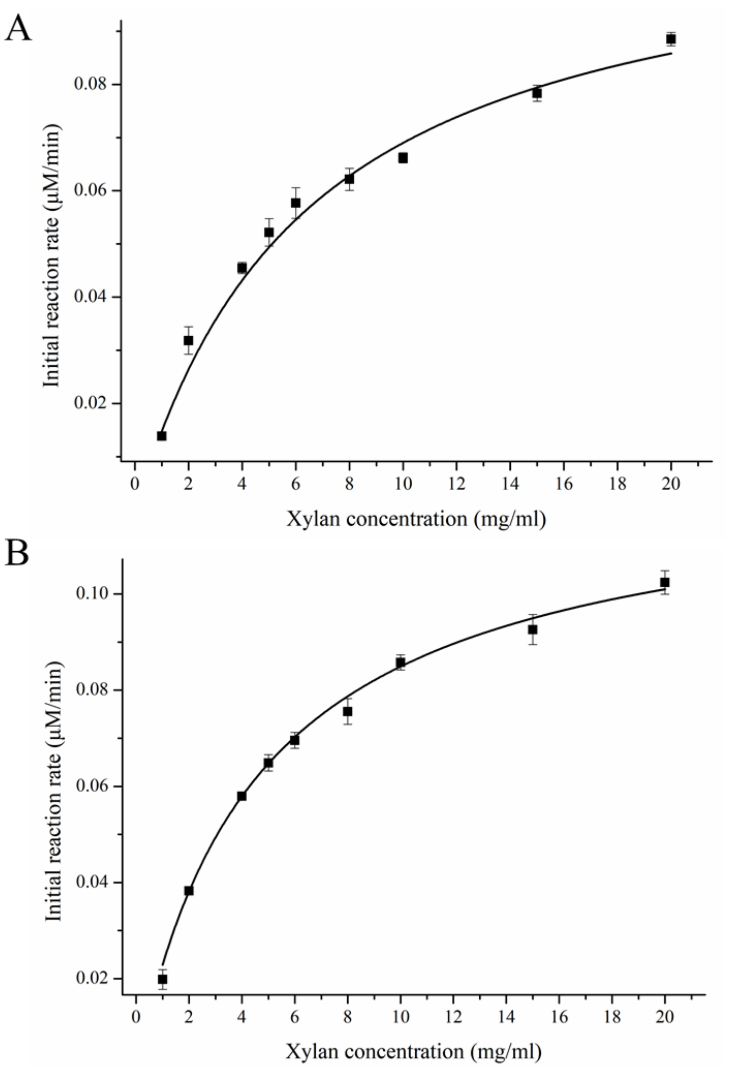

Supplementary Fig. S4 Michaelis-Menten plots for KM values of (A) PW-Xyl9 and (B) PW-Xyl37. The initial reaction velocities were obtained in citrate buffer with pH 7 at optimal temperature with enzyme concentrations of 1.327 µg/ml (PW-Xyl9) and 4.733 µg/ml (PW-Xyl37). Data were fitted using the nonlinear regression by origin 8.5 software. The value represents the mean value of the triplicate experiments, and the error bar is the standard deviation.

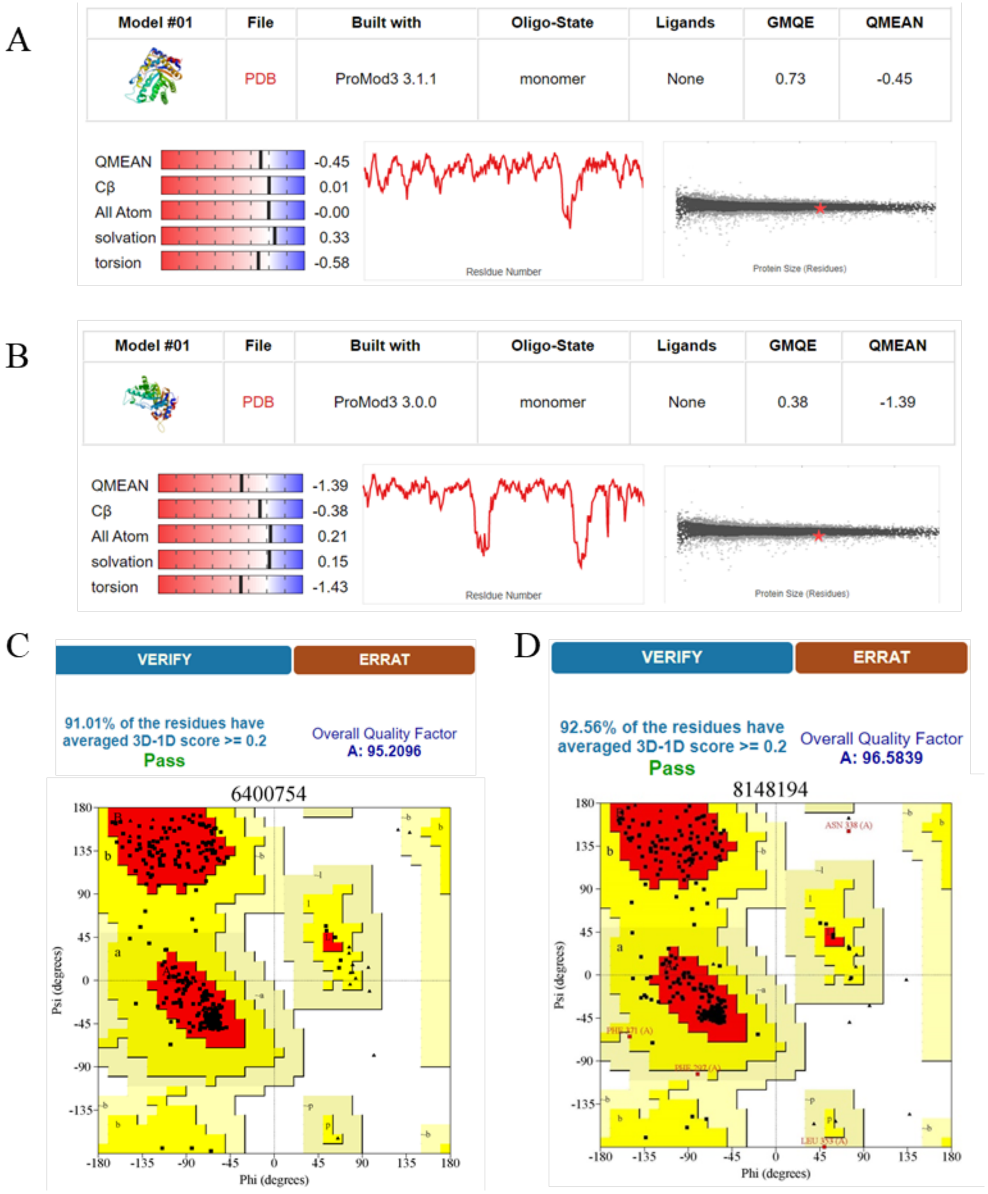

Supplementary Fig. S5 Model evaluation of xylanase genes of PW-xyl9 and PW-xyl37. (A) The evaluation results of PW-xyl9 predicted with SWISS-model. (B) The evaluation results of PW-xyl37 predicted with SWISS-model. (C) and (D) Model quality of PW-xyl9 and PW-xyl37 predicted with SAVES 's evaluation (VERIFY>80% , ERRAT>85%, PROCHECK: more than 90% of the residue should be in red to qualify).
